# Three new species of *Cortinarius* subgenus *Telamonia* (Cortinariaceae, Agaricales) from China

**DOI:** 10.3897/mycokeys.69.49437

**Published:** 2020-07-14

**Authors:** Meng-Le Xie, Tie-Zheng Wei, Yong-Ping Fu, Dan Li, Liang-Liang Qi, Peng-Jie Xing, Guo-Hui Cheng, Rui-Qing Ji, Yu Li

**Affiliations:** 1 Life Science College, Northeast Normal University, Changchun 130024, China Jilin Agricultural University Changchun China; 2 Engineering Research Center of Edible and Medicinal Fungi, Ministry of Education, Jilin Agricultural University, Changchun 130118, China Northeast Normal University Changchun China; 3 State Key Laboratory of Mycology, Institute of Microbiology, Chinese Academy of Sciences, Beijing 100101, China Institute of Microbiology Beijing China; 4 Microbiology Research Institute, Guangxi Academy of Agriculture Sciences, Nanning, 530007, China Guangxi Academy of Agriculture Sciences Nanning China; 5 College of Plant Protection, Shenyang Agricultural University, Shenyang 110866, China Shenyang Agricultural University Shenyang China

**Keywords:** Ectomycorrhizal fungi, morphology, phylogeny, taxonomy

## Abstract

*Cortinarius* is an important ectomycorrhizal genus that forms a symbiotic relationship with certain trees, shrubs and herbs. Recently, we began studying *Cortinarius* in China and here we describe three new species of Cortinarius subg. Telamonia based on morphological and ecological characteristics, together with phylogenetic analyses. *Cortinarius laccariphyllus***sp. nov.** (section Colymbadini) is associated with broadleaf trees, with strongly hygrophanous basidiomata, special *Laccaria*-like lamellae and white and extremely sparse universal veil. *Cortinarius neotorvus***sp. nov.** (section Telamonia) is associated with broadleaf trees and is easily confused with *C. torvus*, but can be distinguished by the colour of the fresh basidiomes and the stipe usually somewhat tapering towards the base. *Cortinarius subfuscoperonatus***sp. nov.** (section Fuscoperonati) is associated with coniferous trees, with subglobose to broadly ellipsoid spores and is closely related to *C. fuscoperonatus*. A key to the new species and similar species in sections *Colymbadini*, *Telamonia* and *Fuscoperonati* is provided.

## Introduction

*Cortinarius* (Pers.) Gray is one of the most species-rich agaric genera, with reportedly more than 2250 species worldwide (He et al. 2019). While most of the *Cortinarius* species were described from Europe and North America, there are also some species described from Oceania (e.g. Bougher and Hilton 1989; Soop 2005; Gasparini and Soop 2008), South America (e.g. Valenzuela and Esteve-Raventos 1994; Garnica et al. 2003; San-Fabian et al. 2018) and Asia (e.g. Miyauchi 2001; Peintner et al. 2003; Xie et al. 2019). It was assumed that more than 900 species occur in northern European countries, based on phylogenetic studies (Niskanen et al. 2012). At least 500 *Cortinarius* species were reported in North America (Bessette et al. 1997). Only 229 *Cortinarius* species have been reported in China (Teng 1963; Tai 1979; Shao and Xiang 1997; Wei and Yao 2013; Li et al. 2015; Xie 2018; Xie et al. 2019; Cheng et al. 2019; Wei and Liu 2019). Recently, many new species have been described, based on the phylogenetic analyses, together with morphological and ecological data (e.g. Bojantchev and Davis 2011; Wei and Yao 2013; Harrower et al. 2015; Brandrud et al. 2018a). Many studies showed that nrDNA ITS barcodes are typically effective in distinguishing *Cortinarius* species (e.g. Liimatainen et al. 2014; Garnica et al. 2016; Schmidt-Stohn et al. 2017; Brandrud et al. 2018b).

Previously, phylogenetic studies of *Cortinarius* have shown that many traditional infrageneric groups are artificial (Høiland & Holst-Jensen 2000; Garnica et al. 2003; Garnica et al. 2005; Niskanen 2008; Harrower et al. 2011), based on ITS+LSU datasets or only ITS datasets. Cortinarius subg. Telamonia (Fr.) Trog sensu lato was a traditional subgenus, based on moist to dry, strongly to weakly hygrophanous and often brown coloured pileus (Brandrud et al. 1989; Bidaud et al. 1994). Some species in traditional subgenus Telamonia sensu lato were classified into other subgenera and several new sections in subgenus Telamonia sensu stricto, based on phylogenetic studies (Niskanen et al. 2015; Soop et al. 2019). Sections *Colymbadini* Melot, *Fuscoperonati* Liimat. & Niskanen and *Telamonia* (Fr.) Gillot & Lucand, all belonging to subgenus Telamonia sensu stricto, are included in this paper.

The diverse ecosystems in China provide a conducive environment for the growth of *Cortinarius* species. Research, dedicated to the phylogeny and taxonomy of Chinese *Cortinarius*, was initiated in recent years. During field trips in the past years, many specimens of *Cortinarius* were collected from China. However, only two new *Cortinarius* species have been described and reported, based on Chinese specimens until now (Wei and Yao 2013; Xie et al. 2019). There are still many species that have never been reported according to the phylogenetic analyses, based on our materials. Further efforts are necessary to describe these species and reveal the species diversity of *Cortinarius* in China. In this study, three new species of the subgenus Telamonia sensu stricto were described, based on morphological and ecological characteristics, as well as phylogenetic analyses. An identification key to the new species and similar species in sections *Colymbadini*, *Telamonia* and *Fuscoperonati* is provided.

## Materials and methods

### Sampling and morphological studies

We collected specimens from northeast China and northwest China, two important floristic areas of China. Fresh basidiomata were photographed and noted under daylight in the field, dried in an oven at about 50 °C and deposited in the Herbarium of Mycology, Jilin Agricultural University (HMJAU).

The macroscopic characters were described from fresh basidiomata. Colour codes were taken from Kornerup and Wanscher (1978). The microscopic characters were examined from dried specimens mounted in 5% aqueous potassium hydroxide (KOH) and Melzer’s reagent using a Zeiss AX10 light microscope with a high-resolution 100× objective. Twenty to thirty mature basidiospores were measured (excluding apiculus and ornamentation) from each collection. The length/width ratio (Q) was calculated for individual spores. `X and `Q refer to the average value of basidiospores of each specimen. The basidia (ten basidia per collection), sterile cells of lamellar edge (20 sterile cells per collection) and hyphae of the lamellar trama were examined and measured from the pieces of lamellae. The pileipellis structure was studied from radial sections half-way from the pileus centre. Basidiospores, lamellar margin cells of this new species were photographed.

### DNA extraction, PCR amplification and sequencing

We extracted the DNA from fresh tissue dried in silica gel by the NuClean PlantGen DNA Kit (CWBIO, China) and amplified the ITS region with primers ITS1F and ITS4 (White et al. 1990; Gardes and Bruns 1993).The PCR amplification progress followed Xie et al. (2019) and was sequenced by Sangon Biotech (Shanghai) Co. Ltd. The newly generated ITS sequences have been submitted to GenBank.

### Data analysis

BLAST searches with the newly-generated ITS sequences were performed against NCBI (https://www.ncbi.nlm.nih.gov/) and UNITE (https://unite.ut.ee/) databases to retrieve similar sequences for the phylogenetic analyses (Table 1). *C. armillatus* (Fr.: Fr.) Fr. and *C. paragaudis* Fr. of section Armillati Kühner & Romagn. ex M.M. Moser, Schweiz. Z. Pilzk. were chosen as outgroup. Section Armillati belongs to subgenus Telamonia sensu stricto and is separated from other sections (Niskanen 2008; Niskanen et al. 2011).

**Table 1.** ITS sequences used in the phylogenetic analysis. New species in bold.

Species	Voucher	GenBank accession No.	Locality	Reference
*C. agathosmus* TYPE	CFP536	KC608590	Sweden	Niskanen et al. (2013a)
*C. ahsii* TYPE	MM19650703 (IB)	KX882644	USA	Ammirati et al. (2017)
*C. ahsii*	JFA10303 (WTU)	KX882649	USA	Ammirati et al. (2017)
*C. alboviolaceus*	HMJAU44214	MK552393	China	This study
*C. alboviolaceus*	HMJAU44245	MK234572	China	Xie et al. (2019)
*C. alboviolaceus*	HMJAU44347	MK552392	China	This study
*C. alboviolaceus*	F15809	FJ157005	Canada	Harrower et al. (2011)
*C. armeniacus*	HMJAU44408	MK552394	China	This study
*C. armeniacus*	F16352	FJ039573	Canada	Harrower et al. (2011)
*C. armillatus* TYPE	F256861 (S)	NR131891	Sweden	Kytövuori et al. (2005)
*C. bulliardii*	CFP499 (S)	JX114942	Sweden	Ammirati et al. (2013)
*C. caesioarmeniacus* TYPE	H7000901	KP137498	Canada	Liimatainen (2014)
*C. caesioarmeniacus*	HMJAU44409	MK552396	China	This study
*C. caesioarmeniacus*	HMJAU44403	MK552395	China	This study
*C. cinnabarinus*	IK85-1517 (H)	JX114943	Finland	Ammirati et al. (2013)
*C. cinnabarinus* TYPE	CFP379 (S)	JX114944	Sweden	Ammirati et al. (2013)
*C. coccineus* TYPE	435745 (GK)	JX114945	France	Ammirati et al. (2013)
*C. colynbadinus*	CFP1130 (S)	JX127302	Sweden	Ammirati et al. (2013)
*C. colynbadinus* TYPE	F248443 (S)	NR131819	Sweden	Ammirati et al. (2013)
*C. fructuodorus*	TN09-113	KC608582	USA	Niskanen et al. (2013a)
*C. fructuodorus* TYPE	H7001104	NR131827	USA	Niskanen et al. (2013a)
*C. fuscoperonatus*	SSt16-046	MF139754	Sweden	Schmidt-Stohn et al. (2017)
*C. fuscoperonatus*	CFP1470	JX407330	France	Niskanen et al. (2013b)
*C. fuscoperonatus*	CFP505	EU433390	Sweden	GenBank/Liimatainen
***C. laccariphyllus***	**HMJAU44449**	**MK552380**	**China**	**This study**
***C. laccariphyllus***	**HMJAU44450**	**MK552381**	**China**	**This study**
*C. millaresensis*	XC2011-200	MH784748	France	Bidaud et al. (2017)
*C. millaresensis*	XC2013-163	MH784752	France	Bidaud et al. (2017)
*C. nolaneiformis*	DB886 (BP)	KJ206487	Hungary	Dima et al. (2014)
*C. nolaneiformis* TYPE	PRM857042	NR131833	Czech Republic	Dima et al. (2014)
*C. paragaudis* TYPE	F256858 (S)	NR131814	Norway	Niskanen et al. (2011)
*C. privignofulvus* TYPE	AB00-10-128 (PC)	MH784703	France	Bidaud et al. (2017)
*C. privignofulvus*	AB04-09-192	MH784714	France	Bidaud et al. (2017)
***C. neotorvus***	**HMJAU44438**	**MK552383**	**China**	**This study**
***C. neotorvus***	**HMJAU44441**	**MK552384**	**China**	**This study**
***C. neotorvus***	**HMJAU44442**	**MK552385**	**China**	**This study**
***C. neotorvus***	**HMJAU44443**	**MK552386**	**China**	**This study**
***C. neotorvus***	**HMJAU44437**	**MK552382**	**China**	**This study**
*C. rigidipes* TYPE	MM1962/0062 (IB)	KJ206504	Switzerland	Dima et al. (2014)
*C. rigidipes*	IK95-1873 (H)	KJ206506	Germany	Dima et al. (2014)
*C. subargyronotus* TYPE	H7018127	KP137494	Sweden	Liimatainen (2014)
***C. subfuscoperonatus***	**HMJAU44446**	**MK552389**	**China**	**This study**
***C. subfuscoperonatus***	**HMJAU44447**	**MK552390**	**China**	**This study**
***C. subfuscoperonatus***	**HMJAU44445**	**MK552388**	**China**	**This study**
***C. subfuscoperonatus***	**HMJAU44444**	**MK552387**	**China**	**This study**
***C. subfuscoperonatus***	**HMJAU44448**	**MK552391**	**China**	**This study**
*C. torvus*	TUB 011515	AY669668	Germany	Garnica et al. (2005)
*C. torvus*	IK98-1973	JX407337	Denmark	Niskanen et al. (2013b)
*C. torvus*	TF01-035	AJ889977	Denmark	GenBank/Kjoller
*C. turgidoides*	AB15-09-37	MH784723	France	Bidaud et al. (2017)
*C. turgidoides*	AB07-09-121	MH784717	France	Bidaud et al. (2017)
*C. uraceomajalis*	DB2291 (BP)	KJ206511	Hungary	Dima et al. (2014)
*C. uraceomajalis*	DB2283 (BP)	KJ206510	Hungary	Dima et al. (2014)
*C. uraceomajalis* TYPE	DB1623 (BP)	NR131835	Hungary	Dima et al. (2014)
*C. uraceonemoralis*	ORS-ERDO99-15-1 (BP)	KJ206520	Hungary	Dima et al. (2014)
*C. uraceonemoralis* TYPE	H7017739	NR131836	Italy	Dima et al. (2014)
*C. uraceus* TYPE	TN04-872 (H)	NR131837	Finland	Dima et al. (2014)
*C. uraceus*	IK98-1607 (H)	KJ206525	Finland	Dima et al. (2014)
*C. vernalisierraensis* TYPE	DBB33386 (UC)	KX882652	USA	Ammirati et al. (2017)
*C. vernalisierraensis*	DBB15144 (UC)	KX882653	USA	Ammirati et al. (2017)

All ITS sequences were aligned and edited with BioEdit 7.0.9 (Hall 1999). ITS1 and ITS2 were delimited by comparison with the sequence KC608590, which is fully annotated in GenBank. For phylogenetic analyses, both Bayesian Inference (BI) and Maximum Likelihood (ML) methods were used. The analyses were performed with two partitions, one including ITS1 and ITS2, the other including coding sequences (SSU, 5.8S and LSU). The two partitions alignments were concatenated using Phyutility 2.2 (Smith and Dunn 2008). Exactly identical sequences were removed from the data matrix (Vadthanarat et al. 2017). For BI analysis, the best-fit model for each partition was determined using the Akaike Information Criterion (AIC), implemented in MrModeltest 2.3 (Nylander 2004). BI analysis was performed with MrBayes 3.2.6 (Ronquist and Huelsenbeck 2003). Markov Chain Monte Carlo (MCMC) chains were run for 200,000 generations, sampling every 100th generation at which point the average standard deviation of split frequencies was 0.00594. The first 25% of trees were discarded to build the 50% majority rule consensus tree. ML analysis was performed with RAxML (Stamatakis 2014) and implemented in raxmlGUI (Silvestro and Michalak 2012). All parameters in the ML analysis were kept as defaults, except for choosing GTRGAMMAI as the model of sequence evolution. For testing the support of the branches, rapid bootstrap analysis with 1,000 replicates was chosen. The resulting phylogenies were visualised in FigTree 1.4.3 (http://tree.bio.ed.ac.uk/software/figtree/).

## Results

### Phylogenetic analyses

**Figure 1.**
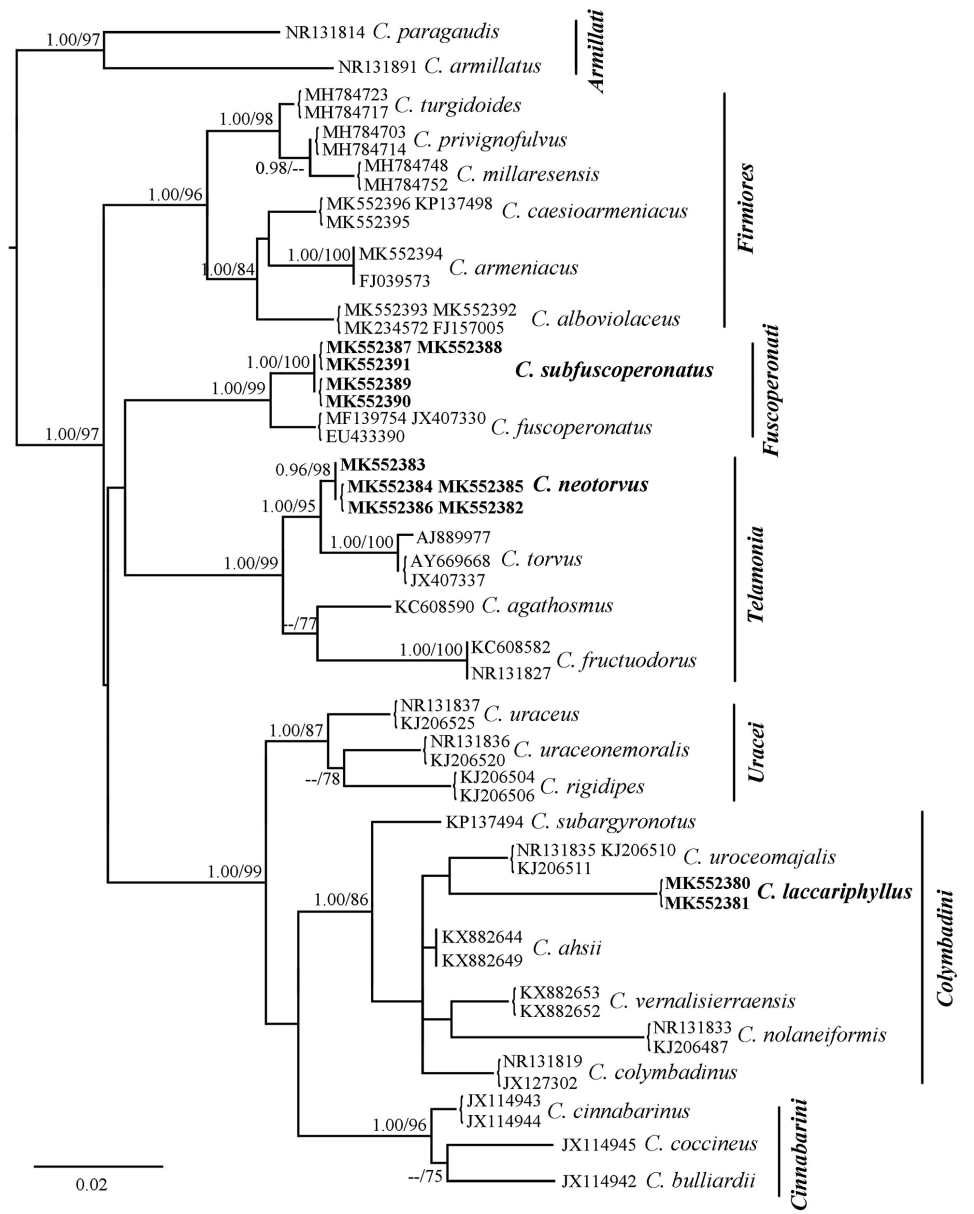
Maximum Likelihood tree inferred from ITS sequences. The tree is rooted with section Armillati. Bayesian posterior probabilities (≥ 0.95) and ML bootstrap values (≥ 75%) are shown on each branch (BPP / ML). New species in bold.

The dataset for phylogenetic analyses contained 60 ITS sequences, representing 27 species (Table 1). The combined matrix of 33 samples with 583 nucleotide sites (including 366 informative sites) is available from TreeBASE under S26123 (study accession URL: http://purl.org/phylo/treebase/phylows/study/TB2:S26123). GTR+G and JC were chosen as the best-fit model for ITS1+ITS2 partition and SSU+5.8S+LSU partition, respectively. The BI and ML trees showed similar topologies with high statistical support values. The ML tree was selected as the representative phylogeny (Fig. 1). The Bayesian posterior probabilities (BPP) ≥ 0.95 and ML bootstrap values (ML) ≥ 75% are shown on the branches.

The phylogenetic analyses recovered seven sections, including outgroup (Fig. 1). Three new species were separated into individual lineages with high statistical support values and were distinct from their closest taxa, respectively. *Cortinarius laccariphyllus* had a distinct position with other species in section Colymbadini (BPP = 1.00, MLBS = 86%). The five collections of *C. neotorvus* (BPP = 0.96, MLBS = 98%) formed a sister relationship (BPP = 1.00, MLBS = 95%) with *C. torvus* (Fr.) Fr. in section Telamonia. *Cortinarius subfuscoperonatus* (BPP = 1.00, MLBS = 100%) formed a sister relationship (BPP = 1.00, MLBS = 99%) with *C. fuscoperonatus* Kühner in section Fuscoperonati.

### Taxonomy

#### Cortinarius laccariphyllus

Taxon classificationFungiAgaricalesCortinariaceae

Y. Li & M.L. Xie sp. nov.

1E5CEDF8-1D56-5202-BD75-4509CF2BC11F

830780

**Figure 2.**
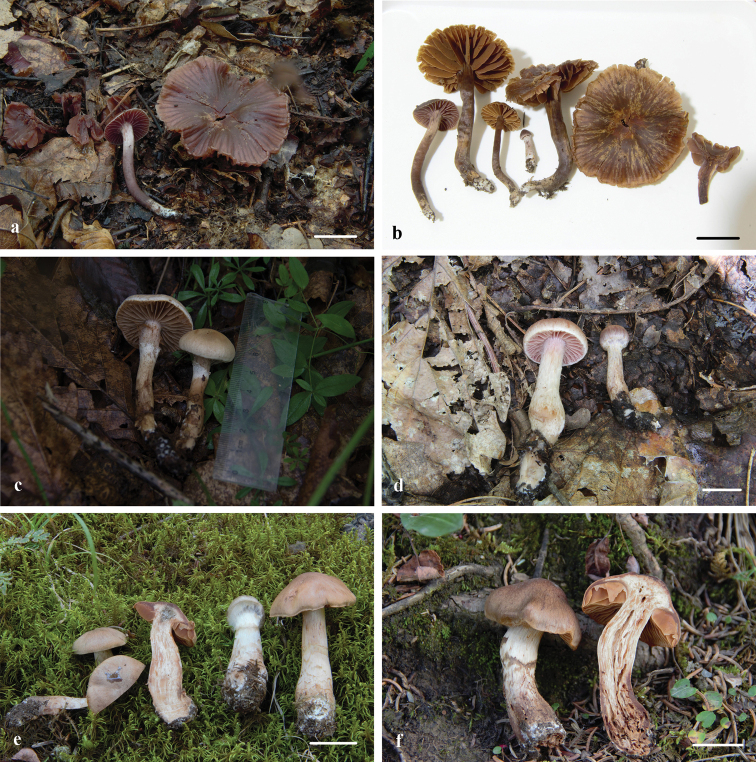
Basidiocarps of three newly-described species. **a, b***Cortinarius laccariphyllus* (**a, b** HMJAU44449, holotype); **c, d***Cortinarius neotorvus* (**c** HMJAU44441, holotype; **d** HMJAU44439); **e, f***Cortinarius subfuscoperonatus* (**e** HMJAU44444, holotype; **f** HMJAU44445). Scale bars: 2 cm (**a, b, d–f**). Photographs by Meng-Le Xie.

**Figure 3.**
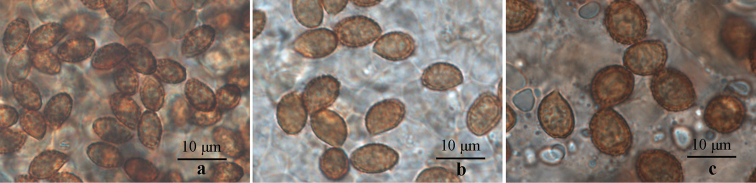
Basidiospores of three newly-described species. **a***Cortinarius laccariphyllus* (HMJAU44449, holotype); **b***Cortinarius neotorvus* (HMJAU44441, holotype); **c***Cortinarius subfuscoperonatus* (HMJAU44444, holotype). Photographs by Meng-Le Xie.

**Figure 4.**
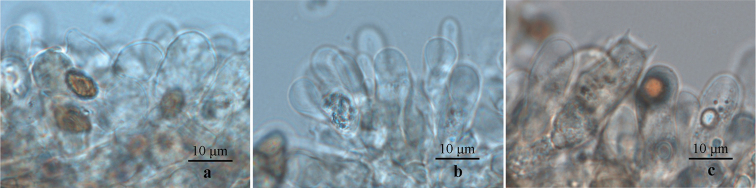
Margin cells of three newly-described species. **a***Cortinarius laccariphyllus* (HMJAU44449, holotype); **b***Cortinarius neotorvus* (HMJAU44441, holotype); **c***Cortinarius subfuscoperonatus* (HMJAU44444, holotype). Photographs by Meng-Le Xie.

Figures 2a, b , 3a , 4a

##### Diagnosis.

Pileus 2.2–6.6 cm in diam., strongly hygrophanous, translucently striate. Lamellae distant, *Laccaria*-like when young. Universal veil white, extremely sparse. Basidiospores 7.7–9.7 × 4.5–5.8 μm. The ITS sequences differ from the sequences of other species of section Colymbadini by at least fifteen substitutions and eight indel positions.

##### Holotype.

China. Jilin Province: Antu County, Liangjiang Town, Dongfanghong Village, broadleaf forest (*Quercus mongolica* dominated forest with some *Juglans* and *Acer*), 42°42'51"N, 128°01'10"E, alt. 640 m, 5 August 2017, M.L. Xie, HMJAU44449, GenBank No. (ITS) MK552380.

##### Etymology.

The name refers to the *Laccaria*-like lamellae when young.

##### Description.

Pileus 2.2–6.6 cm in diam., conical when young, then convex, strongly hygrophanous, reddish-brown (9E6–8), dark brown at the centre (8F6–8), margin to half-way translucently striate, rarely fibrillose, margin thin and wavy. Lamellae subadnate to emarginated, distant, *Laccaria*-like (*Laccaria laccata* (Scop.) Cooke) when young, reddish-brown (9E6–8) to rusty brown (6E8), edge slightly serrate. Stipe 4.2–6.6 cm long, 0.4–0.8 cm thick at apex, 0.2–0.5 cm thick at base, cylindrical to tapering towards base, dark brown (7F6) to black brown (7F3), surface with white fibrillose when young, these disappearing with age (excluding the base of stipe). Universal veil white, extremely sparse, soon disappearing. Context dark brown (7F6–8), strongly hygrophanous (pileus and stipe). Odour indistinct. Exsiccata brown (5F8) to black brown (7F5). UV fluorescence yellow on stipe, pileus and lamellar edge, strong at stipe base.

Basidiospores 7.7–9.7 × 4.5–5.8 μm, Q = 1.43–1.84, `X = 8.0–8.5 × 4.8–5.2 μm, `Q = 1.66–1.70 (60 spores, 2 specimens), ellipsoid to amygdaloid, moderately and sharply verrucose, moderately dextrinoid. Basidia 4-spored, clavate, 23–39 × 7–9 μm, thin-walled, hyaline to olivaceous brown in 5% KOH. Lamellar edge fertile, with cylindrical-clavate sterile cells, 14–41 × 7–17 μm, thin-walled, hyaline in 5% KOH. Lamellar trama hyphae regular, pale olivaceous to olivaceous brown in 5% KOH, finely and densely encrusted. Pileipellis: epicutis hyphae cylindrical, 4–9.5 μm wide, dark olivaceous brown in 5% KOH, encrusted; hypocutis well developed, hyphae 11.5–53 μm wide, sub-cellular to cylindrical, slightly olivaceous in 5% KOH, finely encrusted. Pileus trama hyphae thin-walled, hyaline to slightly olivaceous in 5% KOH, smooth to finely encrusted. Clamp connections present.

##### ITS sequence.

The ITS sequences of two specimens are 534 bp long and 100% identical. They differ from the sequences of other species of section Colymbadini (Niskanen et al. 2013a; Dima et al. 2014; Ammirati et al. 2017) by at least fifteen substitutions and eight indel positions.

##### Ecology and distribution.

In broadleaf forest (*Quercus mongolica* dominated forest). Gregarious. Known from Jilin Province, China.

##### Additional specimens examined.

China. Jilin Province: Antu County, Liangjiang Town, Dongfanghong Village, broadleaf forest (*Quercus mongolica* dominated forest with some *Juglans* and *Acer*), 42°42'51"N, 128°01'10"E, alt. 640 m, 5 August 2017, M.L. Xie, HMJAU44450, GenBank No. (ITS) MK552381.

##### Comments.

*Cortinarius laccariphyllus* has strongly hygrophanous basidiomata, *Laccaria*-like (when young), with distantly-spaced lamellae and an extremely sparse, white veil. Morphologically, *C. nolaneiformis* (Velen.) Dima, Niskanen & Liimat. is similar to *C. laccariphyllus* due to the strongly hygrophanous pileus, similar colouration and similar size of spores. *Cortinarius uraceomajalis* Dima, Liimat., Niskanen & Bojantchev is also similar to *C. laccariphyllus* because of the black brown stipe and the striate pileus. However, both *C. nolaneiformis* and *C. uraceomajalis* have a yellowish veil and medium-spaced lamellae and lamellae not *Laccaria*-like. Furthermore, *C. nolaneiformis* is associated with broadleaf trees and also occurs in coniferous forest; *C. uraceomajalis* has a somewhat lighter brown pileus as well as generally smaller (av. 7.8–8.1 × 4.6–4.7 μm) and narrower (Qav. > 1.7) spores (Dima et al. 2014). In the phylogenetic analyses, *C. laccariphyllus* was well separated from other species in section Colymbadini.

#### Cortinarius neotorvus

Taxon classificationFungiAgaricalesCortinariaceae

Y. Li, M.L. Xie & T.Z. Wei sp. nov.

BD6A31B1-0337-5FC5-A8B9-EA30AE5B3ABB

835346

Figures 2c, d , 3b , 4b

##### Diagnosis.

Pileus 2–4.4 cm in diam., weakly hygrophanous, orange grey. Lamellae greyish-red when young. Stipe cylindrical to somewhat tapering towards base. Universal veil greyish-yellow. Context white, sometimes with violet tinge at the stipe apex. Basidiospores 8.5–10.2 × 5.8–6.9 μm. Lamellar edge sterile. The ITS sequence of the holotype differs from the sequences of other species in section Telamonia by at least six substitutions and five indels.

##### Holotype.

China. Jilin Province: Antu County, Liangjiang Town, Dongfanghong Village, broadleaf forest (*Quercus mongolica* dominated forest with some *Juglans* and *Acer*), 42°42'51"N, 128°01'10"E, alt. 640 m, 5 August 2017, M.L. Xie, HMJAU44441, GenBank No. (ITS) MK552384.

##### Etymology.

The name refers to *Cortinarius torvus*.

##### Description.

Pileus 2–4.4 cm in diam., hemispherical when young, then convex to almost plane with a low, broad umbo, weakly hygrophanous, orange grey (5B2), paler at the margin, surface with greyish-white fibrillose. Lamellae emarginate, medium-spaced, yellowish-grey (4B2), greyish-red (9B4–6) when young, sometimes with violet tinge when young, margin paler, slightly serrate. Stipe 4.1–10.5 cm long, 0.5–0.7 cm thick at apex, 0.3–0.5 cm thick at base, cylindrical to somewhat tapering towards base, orange grey (5B2) when moist, sometimes with violet tinge at the apex when young, surface with richly whitish fibrillose. Universal veil greyish-yellow (4B3), copious, usually forming a girdle on the upper stipe, cortina white. Context white (A1), marbled watery when moist, sometimes with violet tinge at the apex of the stipe. Odour indistinct. Exsiccata brown (6E5) to dark brown (6F6).

Basidiospores 8.5–10.2 × 5.8–6.9 μm, Q = 1.31–1.67, `X = 9.0–9.9 × 6.1–6.5 μm, `Q = 1.45–1.61 (130 spores, 6 collections), ellipsoid, moderately verrucose, moderately dextrinoid. Basidia 4-spored, cylindrical to clavate, 27–53 × 7–12 μm, thin-walled, hyaline to olivaceous brown in 5% KOH. Lamellar edge sterile, sterile cells cylindrical-clavate, 11–26 × 3–9 μm, thin-walled, hyaline in 5% KOH. Lamellar trama hyphae regular, pale olivaceous in 5% KOH, smooth. Universal veil hyphae thin-walled, hyaline to pale olivaceous yellow in 5% KOH. Pileipellis: epicutis hyphae cylindrical, 2–6 μm wide, olivaceous brown in 5% KOH, smooth; hypocutis well developed, hyphae 15–38 μm wide, sub-cellular, thin-walled, hyaline in 5% KOH, smooth. Pileus trama hyphae thin-walled, hyaline to slightly olivaceous in 5% KOH, smooth. Clamp connections present.

##### ITS sequence.

The ITS sequences of *C. neotorvus* are 513–515 bp long (5 collections, Table 1). All four sequences (MK552384 holotype, MK552385, MK552386 and MK552382) are identical and only MK552383 has 2 bp indels. The ITS sequence of *C. neotorvus* (MK552384, holotype) differs from the sequences of other species in section Telamonia by at least six substitutions and five indels.

##### Ecology and distribution.

In broadleaf forest (*Quercus mongolica* dominated forest). Solitary or gregarious. Known from Jilin and Heilongjiang Province, China.

##### Additional specimens examined.

China. Heilongjiang Province: Heihe City, Wudalianchi Scenic Area, broadleaf forest (*Quercus mongolica*), 48°39'15"N, 126°28'18"E, alt. 290 m, 16 August 2017, M.L. Xie, HMJAU44442, GenBank No. (ITS) MK552385; 12 August 2018, P.J. Xing, HMJAU44440; Heihe City, Shengshan National Nature Reserve, broadleaf forest (*Quercus mongolica* dominated forest with some *Tilia* and *Alnus*), 49°30’N, 126°43’E, alt. 300 m, 11 September 2017, G.H. Cheng, HMJAU44443, GenBank No. (ITS) MK552386. Jilin Province: Yanji City, Sandaowan Town, broadleaf forest (*Quercus mongolica*), 43°16'10"N, 129°07'19"E, alt. 580 m, 8 September 2018, M.L. Xie, HMJAU44437, GenBank No. (ITS) MK552382, HMJAU44438, GenBank No. (ITS) MK552383, HMJAU44439.

##### Comments.

*Cortinarius neotorvus* is easily confused with *C. torvus* due to highly similar morphology. Morphologically, the lamellae of *C. torvus* are adnate to subdecurrent and distant (Bidaud et al. 1999; Breitenbach and Kränzlin 2000; Soop 2014) and the pileus colour of *C. torvus* is usually darker and with a violet tinge, as well as the stipe usually being bulbous at the base (Consiglio et al. 2003). In molecular data, the ITS sequence of *C. neotorvus* (MK552384, holotype) differ from the sequences of *C. torvus* (AY669668, JX407337) by six substitutions and five indels. In the phylogenetic analyses, the five specimens of *C. neotorvus* were placed in separate monophyletic lineages (BPP = 0.96, MLBS = 90%) and formed a sister relationship with *C. torvus*.

#### Cortinarius subfuscoperonatus

Taxon classificationFungiAgaricalesCortinariaceae

Y. Li & M.L. Xie sp. nov.

49ACC408-1D4C-5D7E-8B08-9D7616BF2C43

830782

Figures 2e, f , 3c , 4c

##### Diagnosis.

Pileus 1.6–4.4 cm in diam. Context white, greyish-brown when moist. Basidiospores 9.5–12.1 × 7.9–9.7 μm. The ITS sequence of the holotype differs from other species in section Fuscoperonati by at least six substitutions and six indels.

##### Holotype.

China. Gansu Province: Zhangye City, Minle County, Gansu Qilianshan National Nature Reserve, coniferous forest (*Picea crassifolia*), 38°17'55"N, 100°45'54"E, alt. 2860 m, 9 August 2018, M.L. Xie, HMJAU44444, GenBank No. (ITS) MK552387.

##### Etymology.

The name refers to its affinity to *Cortinarius fuscoperonatus*.

##### Description.

Pileus 1.6–4.4 cm in diam., hemispherical when young, then low convex, weakly hygrophanous, pale greyish-brown (6C3), sometimes reddish-brown (9E5–9E6) to dark brown (6F6–6F8), surface with greyish-brown fibrillose, margin wavy with age. Lamellae emarginate, medium-spaced, reddish-brown to rusty brown (7D6–7E7), margin even when young, then slightly serrate. Stipe 2.3–7.5 cm long, 0.8–1.3 cm thick at apex, 1.5–2.5 cm thick at base, clavate, white to pale grey (E2), mycelium white at the base. Universal veil greyish-brown (6C2), rich, usually forming an annular band on the middle part and distinct belts or zones lower down. Context white (A1), greyish-brown (7F8) and marbled watery when moist, strongly hygrophanous near pileus and lamellae. Odour somewhat radish-like. Chemical reaction: pileus and context (fresh basidiomata) are dark black brown (8F3) with 10% KOH. Exsiccata brown (6E5) to dark brown (6F5).

Basidiospores 9.5–12.1 × 7.9–9.7 μm, Q = 1.10–1.45, `X = 10.3–11.2 × 8.0–8.6 μm, `Q = 1.24–1.33 (135 spores, 5 collections), subglobose to broadly ellipsoid, moderately to strongly verrucose, strongly dextrinoid. Basidia 4-spored, clavate, 35–58 × 10–13 μm, thin-walled, hyaline to olivaceous brown in 5% KOH. Lamellar edge fertile, with cylindrical-clavate sterile cells, 13–27 × 6–11 μm, thin-walled, hyaline to slightly olivaceous yellow in 5% KOH. Lamellar trama hyphae regular, pale olivaceous to olivaceous brown in 5% KOH, smooth. Pileipellis: epicutis hyphae cylindrical, 4–12 μm wide, slightly olivaceous brown to olivaceous brown in 5% KOH, some hyphae finely encrusted; hypocutis well developed, hyphae 24–89 × 15–29 μm, sub-cellular to sub-cylindrical, thin-walled, hyaline to slightly olivaceous brown in 5% KOH, smooth. Pileus trama hyphae almost thin-walled, hyaline in 5% KOH, smooth. Clamp connections present.

##### ITS sequence.

The ITS sequences of *C. subfuscoperonatus* are 524–525 bp long (5 collections, Table 1) and distinct from other members of section Fuscoperonatus. The ITS sequence of *C. subfuscoperonatus* (MK552387, holotype) differs from *C. fuscoperonatus* by six substitutions and six indels.

##### Ecology and distribution.

In coniferous forest (*Picea crassifolia* dominated forest). Solitary or gregarious. Known from Gansu Province, China.

##### Additional specimens examined.

China. Gansu Province: Zhangye City, Minle County, Gansu Qilianshan National Nature Reserve, coniferous forest (*Picea crassifolia*), 38°17'55"N, 100°45'54"E, alt. 2860 m, 9 August 2018, M.L. Xie, HMJAU44445, GenBank No. (ITS) MK552388; Zhangye city, Su’nan Yugu Autonomous County, Gansu Qilianshan National Nature Reserve, coniferous forest (*Picea crassifolia* dominated forest, occasionally with *Juniperus*), 38°44'57"N, 99°47'56"E, alt. 3010 m, 10 August 2018, M.L. Xie, HMJAU44446, GenBank No. (ITS) MK552389, HMJAU44447, GenBank No. (ITS) MK552390; Zhangye city, Su’nan Yugu Autonomous County, Gansu Qilianshan National Nature Reserve, coniferous forest (*Picea crassifolia* dominated forest, occasionally with *Juniperus*), 38°33'13"N, 100°41'75"E, alt. 2700 m, 11 August 2018, M.L. Xie, HMJAU44448, GenBank No. (ITS) MK552391.

##### Comments.

*Cortinarius subfuscoperonatus* corresponds well to the characteristics of section Fuscoperonati, with weak hygrophanous pileus, an annular band on the middle stipe and distinct belts or zones lower down, large spores (> 10 µm long) and grow in coniferous forests. *Cortinarius fuscoperonatus* was previously placed in section Bovini M.M. Moser (Bidaud et al. 2009; Soop 2014) and *Armillati* (Brandrud et al. 1992), until Niskanen et al. (2015) placed it in section Fuscoperonati. *Cortinarius subfuscoperonatus* has remarkably similar morphological characteristics to *C. fuscoperonatus*, apart from the spores of *C. fuscoperonatus* being narrower (9.7–11.6 × 6.6–7.7 µm), the pileus being chocolate brown to blackish-brown and being fine fibrous to fine scaly (Schmidt-Stohn et al. 2017). In addition, *C. subfuscoperonatus* formed a sister relationship with *C. fuscoperonatus* and was well separated according to the phylogenetic analyses. *C. subfuscoperonatus* could be considered as the second species in section Fuscoperonati.

### Key to new species and morphologically-similar species in sections *Colymbadini*, *Telamonia* and *Fuscoperonati*

**Table:** 

1	Basidiomata medium. Pileus more or less brown, strongly hygrophanous. Stipe usually cylindrical. Universal veil sparse. With positive yellow UV reaction. Associated with coniferous and/or broadleaf trees. Spores ellipsoid to amygdaloid	(**section Colymbadini**) **2**
–	Basidiomata medium to large. Pileus more or less brown and hygrophanous. Stipe cylindrical to clavate. Universal veil white to greyish-yellow, sometimes with violet tinge, usually forming a ring at the middle stipe. Associated with coniferous and/or broadleaf trees	(**section Telamonia**) **3**
–	Basidiomata medium to large. Pileus brown and weakly hygrophanous. Stipe clavate to slightly bulbous. Universal veil greyish-brown to blackish-brown. Associated with coniferous trees. Spores subglobose to ellipsoid, moderately to strongly verrucose	(**section Fuscoperonati**) **4**
2	Pileus strongly hygrophanous, reddish-brown to dark brown, surface translucently striate. Lamellae distant, *Laccaria*-like when young. Stipe cylindrical to tapering towards base, hollow. Universal veil white, extremely sparse. Odour indistinct. Positively yellow UV fluorescence (exsiccata). Associated with broadleaf trees. Spores ellipsoid to amygdaloid, on average 8.0–8.5 × 4.8–5.2 μm.	***C. laccariphyllus***
–	Pileus strongly hygrophanous, yellowish-brown to brown, margin striate. Lamellae medium-spaced. Stipe cylindrical to tapering towards base, not hollow. Universal veil yellow, very sparse. Odour similar to raw vegetables. Usually yellow UV fluorescence at stipe. Associated with broadleaf trees. Spores amygdaloid to narrowly amygdaloid, on average 7.8–8.1 × 4.6–4.7 μm	***C. uraceomajalis***
–	Pileus strongly hygrophanous, dark greyish-brown to dark brown, margin slightly striate. Lamellae medium-spaced to fairly distant, margin whitish when young. Stipe cylindrical to clavate, sometimes tapering downwards, sometimes hollow. Universal veil yellow. Strong yellow UV fluorescence at stipe, dull yellowish-brown at pileus, lamellae and context. Associated with coniferous and broadleaf trees. Spores amygdaloid to weakly ellipsoid, on average 8.1–8.6 × 4.8–5.1 μm	***C. nolaneiformis***
3	Pileus pale greyish-yellow, paler at the margin, weakly hygrophanous. Lamellae emarginate, medium-spaced, greyish-red when young, sometimes with violet tinge. Stipe cylindrical to somewhat tapering towards base, pale greyish-yellow. Odour indistinct. Spores ellipsoid, on average 9.0–9.9 × 6.1–6.5 μm	***C. neotorvus***
–	Pileus greyish-brown to chestnut brown, usually with violet tinge at the margin, weakly hygrophanous. Lamellae adnate, subdecurrent to distant, greyish-brown, with violet tinge. Stipe clavate, usually bulb at the base. Odour acidulous. Spores ellipsoid, 8–10.5 × 6–7 μm	***C. torvus***
4	Pileus pale greyish-brown, sometimes reddish-brown to dark brown, margin wavy with age, with greyish-brown fibrillose. Stipe clavate. Spores subglobose to broadly ellipsoid, on average 10.3–11.2 × 8.0–8.6 μm	***C. subfuscoperonatus***
–	Pileus chocolate brown to blackish-brown, pale greyish-brown at the edge, fine fibrous to fine scaly. Stipe clavate, with a bulb at the base. Spores ellipsoid to broadly ellipsoid, 9.7–11.6 × 6.6–7.7 μm	***C. fuscoperonatus***

## Discussion

*Cortinarius* is the most species-rich genus of Agaricales, with most of the described species distributed in the Northern Hemisphere. However, so far, little has been done on *Cortinarius* taxonomy in north-eastern Asia or even in the whole of Asia, leaving an important gap in our knowledge of this genus (Horak 1983). The flora of northern China has a strong affinity shared with the circumboreal areas of Europe and western North America but also harbours some floristic elements with a tropical and subtropical affinity (Wu 1979). Some *Cortinarius* species in northern China are the same as those in Europe and western North America (e.g. Xie 2018; Cheng et al. 2019; Wei and Liu 2019). However, there are also some endemic species in China (Wei and Yao 2015; Xie et al. 2019). Thus far, only 229 *Cortinarius* species (about 10% in the world) have been reported in China. Therefore, studies focusing on Chinese *Cortinarius* are needed.

In this study, we described the phylogenetic relationships amongst the three new species and other species, based on the ITS sequences. However, multiple genes should be used in future studies to describe more complex phylogenetic relationships in *Cortinarius*, which some mycologists have conducted. Peintner et al. (2002) assessed the phylogenetic relationships of *Rozites*, *Cuphocybe* and *Rapacea* by molecular phylogenetic approaches, based on ITS and LSU. Frøslev et al. (2005) analysed the phylogeny of Cortinarius subgenus Phlegmacium, a taxonomically difficult group, based on ITS, RPB1 and RPB2. They speculated that the sequences from RNA polymerase II genes have the potential for resolving the phylogenetic problems of *Cortinarius*. Later, the study of Frøslev et al. (2007) showed that the delimitation of species, based on ITS sequences, is more consistent with a conservative morphological species concept and there is considerable potential for using ITS sequence data as a barcode for section Calochroi. Soop et al. (2019) studied the global supraspecific taxonomy of *Cortinarius* by the phylogenetic approach, based on ITS, LSU, RPB1 and RPB2. Both ITS and LSU datasets and ITS, LSU, RPB1 and RPB2 datasets showed satisfactory results. Although phylogenetic analyses of *Cortinarius* have made significant progress in Europe, North America and even in Australasia, few phylogenetic analyses of *Cortinarius*, based on Chinese materials have been carried out. According to our analysis of ITS data, there are presently less than 200 accessions (excluding sequences obtained from mycorrhiza) from China in GenBank. Thus, the dedicated collection of specimens and studying the phylogeny of *Cortinarius*, based on the ITS or, preferably, multiple genes, are important contributions to the global phylogenetic framework of *Cortinarius*.

## Supplementary Material

XML Treatment for Cortinarius laccariphyllus

XML Treatment for Cortinarius neotorvus

XML Treatment for Cortinarius subfuscoperonatus
